# Flooding of the apoplast is a key factor in the development of hyperhydricity

**DOI:** 10.1093/jxb/ert315

**Published:** 2013-10-11

**Authors:** Niels van den Dries, Sergio Giannì, Anna Czerednik, Frans A. Krens, Geert-Jan M. de Klerk

**Affiliations:** ^1^Wageningen UR Plant Breeding, Droevendaalsesteeg 1, 6700 AA Wageningen, The Netherlands; ^2^Dipartimento Scienze Agrarie e Forestali, Palermo University, Viale delle Scienze 11, 90128 Palermo, Italy; ^3^Plant Ecophysiology, Institute of Environmental Biology, Utrecht University, Sorbonnelaan 16, 3584 CA Utrecht, The Netherlands

**Keywords:** Apoplast, *Arabidopsis thaliana*, gas exchange, hyperhydricity, hypoxia, stomatal aperture, water accumulation.

## Abstract

The physiological disorder hyperhydricity occurs frequently in tissue culture and causes several morphological abnormalities such as thick, brittle, curled, and translucent leaves. It is well known that hyperhydric shoots are characterized by a high water content, but how this is related to the abnormalities is not clear. It was observed that water accumulated extensively in the apoplast of leaves of hyperhydric *Arabidopsis* seedlings and flooded apoplastic air spaces almost completely. In hyperhydric *Arabidopsis* seedlings, the volume of apoplastic air was reduced from 85% of the apoplast to only 15%. Similar results were obtained with hyperhydric shoots of statice. The elevated expression of hypoxia-responsive genes in hyperhydric seedlings showed that the water saturation of the apoplast decreased oxygen supply. This demonstrates a reduced gas exchange between the symplast and its surroundings, which will consequently lead to the accumulation of gases in the symplast, for example ethylene and methyl jasmonate. The impairment of gas exchange probably brings about the symptoms of hyperhydricity. Interestingly, stomatal aperture was reduced in hyperhydric plants, a previously reported response to injection of water into the apoplast. Closure of the stomata and the accumulation of water in the apoplast may be the reasons why seedlings with a low level of hyperhydricity showed improved acclimatization after planting into soil.

## Introduction

Tissue culture has become a powerful and indispensable tool in agriculture and horticulture and is used for breeding, vegetative propagation, and freeing plants from diseases. For plants, the conditions during tissue culture are unnatural and extreme, which may lead to physiological disorders, among others hyperhydricity (HH) (Debergh *et al.*, 1981, 1992; Rojas-Martinez *et al.*, 2010). By reducing the quality and multiplication rate, HH seriously affects commercial micropropagation. Hyperhydric shoots are characterized by various malformations visible to the naked eye such as thick, brittle, curled, and translucent leaves (Gaspar *et al.*, 1995; Saher *et al.*, 2005). Microscopic observations have revealed that leaves of hyperhydric plants have a poorly developed epicuticular wax layer, a reduced number of palisade cells, and large intercellular spaces in the mesophyll (Olmos and Hellin, 1998; Picolli *et al*., 2001; Jausoro *et al.*, 2010). Other abnormalities such as chlorophyll deficiency (Franck *et al.*, 1998), low lignification (Kevers *et al.*, 1987), and malformed stomata (Apostolo and Llorente, 2000) have been reported. HH occurs in a wide variety of plant species, ranging from garlic (Wu *et al.*, 2009) to apple (Chakrabarty *et al.*, 2005). In *Arabidopsis*, HH has been researched only once. A mutant (c*ri1*) developed severe HH *in vitro* when cultured on the commonly used agar concentration of 0.7% (Delarue *et al.*, 1997).

As the naming of the disorder indicates, the water content of hyperhydric plants is high. Affected plants are incapable of maintaining a correct water balance and accumulate water (Rojas-Martinez *et al.*, 2010). The high relative humidity in the culture vessel and the type and concentration of gelling agent are major contributors to the development of HH (Debergh *et al.*, 1992; Ivanova and Van Staden, 2011). Other factors, such as ethylene (Park *et al.*, 2004) and cytokinins (Kadota and Niimi, 2003), may cause HH. Ziv (1991) listed several other factors that influence HH. Two subcellular localizations of the extra water have been proposed. It has been suggested that because of deficiency in both cellulose and lignin, hyperhydric plants have reduced wall pressure and that therefore the symplast takes up more water (Kevers and Gaspar, 1986; George, 1996). Using nuclear magnetic resonance imaging and scanning electron microscopy, Gribble *et al.* (1996, 1998) showed that hyperhydric plants have an excess of water in another location, the apoplast. The apoplast is defined as the cell wall continuum and the intercellular spaces in a plant (Evert, 2006) for a review on the terminology, see Canny, 1995). Water was observed in particular in the intercellular spaces. Accumulation in the apoplast may cause major physiological disorders by disrupting gas exchange within plant tissues (Gribble *et al.*, 2003), since the diffusion rate of gases is 10 000 times slower in water than in air (Jackson, 1985). A causal connection between extra water in the symplast and HH symptoms is not evident at first sight.

To date, many papers have been published on HH, but only a few deal with the underlying mechanisms, and the phenomenon HH is still very poorly understood (Ziv, 1991; Gribble *et al.*, 2003; Rojas-Martinez *et al.*, 2010). Because of the indications that the extra water and the supposed concurrent decrease in air in the apoplast are crucial, and because the volumes of apoplastic water and air have never been adequately quantified in hyperhydric plants, both were measured here using well established methods, mild centrifugation (Terry and Bonner, 1980) and a pycnometric method (Van Noordwijk and Brouwer, 1988), respectively. It was found that in non-hyperhydric plants, air filled 85% of the apoplast and that this air was almost completely replaced by water in hyperhydric plants. In addition, some consequences of excessive water accumulation in the apoplastic space were investigated. Preliminary results have been presented during a congress on *In Vitro* Culture and Horticultural Breeding in Ghent (van den Dries *et al.*, 2012).

## Materials and methods

### Plant growth and treatments

*Arabidopsis thaliana* (Col-0) seeds were sterilized with 70% (v/v) ethanol for 1min, 2% (w/v) sodium hypochlorite for 15min, and subsequently rinsed three times for 10min with sterilized distilled water. Sterile seeds were transferred to a Petri dish with half-strength Murashige and Skoog (MS) basal salt mixture including vitamins (Murashige and Skoog, 1962) supplemented with 1.5% (w/v) sucrose and solidified with 0.7% (w/v) Micro-agar (all from Duchefa Biochemie, Haarlem, The Netherlands). Seeds were stratified in the dark for 3 d at 4 °C and after that germinated in a growth chamber with 16h light/8h dark (30 μmol m^–2^ s^–1^, Philips TL33) at 21 °C. To induce HH, 7-day-old seedlings were transferred to high-sided Petri dishes (nine seedlings per dish) containing the same nutrient medium solidified with 0.2% (w/v) Gelrite (Duchefa Biochemie). Control seedlings were transferred to fresh nutrient medium solidified with 0.7% (w/v) Micro-agar.

Acclimatization to *ex vitro* conditions was studied by transplanting *Arabidopsis* seedlings to soil without the usual precautions for tissue-cultured plants. Thus, an initial period at a high relative humidity was omitted. Transplanted seedlings were grown with 16h light/8h dark (30 μmol m^–2^ s^–1^, Philips TL33) at 20 °C and 70% relative humidity.

Cultures of statice (*Limonium sinuatum*) were kindly provided by Royal van Zanten (Rijsenhout, The Netherlands). Plantlets were grown on medium containing full MS salts, 3% (w/v) sucrose, 0.44 μM 6-benzylaminopurine, and 0.7% (w/v) Daishin agar (Duchefa Biochemie). At subculturing (once per 4 weeks), clumps were separated from one another and placed individually on fresh medium. The cultures were kept in a growth chamber at 21 °C with 16h light/8h dark (30 μmol m^–2^ s^–1^, Phillips TL 33). HH was induced by using 0.2% (w/v) Gelrite instead of 0.7% (w/v) Daishin agar.

### Estimation of apoplastic water and air volumes in leaves

Apoplastic water was extracted from leaf tissues by mild centrifugation (Terry and Bonner *et al.*, 1980). Leaves were excised from plants, weighed, and placed into a spin mini filter microcentrifuge tube (Starlab, Ahrensburg, Germany). Leaves were centrifuged at 3000 *g* for 20min at 4 °C. Immediately after centrifugation, the leaves were reweighed. The presence of symplastic contamination in the apoplastic water was assessed by a malate dehydrogenase (MDH) assay (see below). The apoplastic water volume (V_water_) in μl g^–1^ fresh weight (FW) was calculated using the formula: V_water_=[(FW–W_ac_)×ρH_2_O]/FW. Where FW=fresh weight of leaves in mg, W_ac_=weight of leaves after centrifugation and ρH_2_O=water density (the water density was taken as equal to 1g ml^–1^).

The volume of apoplastic air in leaves was estimated using a pycnometer with a stopper (Raskin, 1983). Leaves were excised, weighed, and placed into the pycnometer. The pycnometer was then filled with distilled water and stoppered. Excess water on the exterior of the pycnometer was removed with filter paper. The weight of the full pycnometer, including leaves, was measured, and the pycnometer (with water and leaves) was subjected to a vacuum (500 mmHg) for 5min to remove air out of the leaves and replace it by water. When required, the vacuum treatment was repeated until all air was removed from the apoplast and the leaves had sunk to the bottom of the pycnometer. After vacuum infiltration, the pycnometer was refilled, dried, and reweighed. The apoplastic air volume (V_air_) in μl g^−1^ FW was calculated using the following formula: V_air_=[(W_bv_–W_av_)×ρH_2_O]/FW. Where W_bv_=weight in mg of the pycnometer including leaves and water before vacuum infiltration, W_av_=weight of the pycnometer including leaves and water after vacuum infiltration, FW=fresh weight of leaves, and ρH_2_O=water density.

### Determination of malate dehydrogenase activity

The presence of cytoplasmic contamination in the extracted apoplastic water was assessed by measuring the activity of MDH. Leaves of hyperhydric seedlings were centrifuged for 20min at 1000, 3000, 7000, 10 000, 15 000, or 21 000 *g*. The fluid, collected after each run, was assayed for MDH activity. The MDH activity was measured in a 1ml reaction mixture containing 0.2mM NADH, 2mM oxaloacetate, and 25 μl of the collected water in 100mM phosphate buffer (pH 7.4). The oxidation of NADH was monitored by measuring the decrease of OD_340_ for 10min. To determine the total activity of MDH in whole-leaf homogenates, leaf cells were disrupted by grinding leaves using a mortar and pestle. Cytoplasmic fluid was collected by centrifugation at 21 000 *g* for 10min. The MDH activity in the apoplastic water was expressed as the percentage of total MDH activity.

### Measurement of water loss rates and relative water contents

The water loss rate was measured 10 d after transfer of seedlings to Gelrite or agar media according to Zhang *et al.* (2011) with minor modifications. Seedlings, including roots, were taken from Petri dishes and the FW was measured. Seedlings were then allowed to desiccate at 22 °C and 48% relative humidity, and were weighed at designated time points during a 180min period (FWt). The water loss rate (WLR) was calculated according to the formula: WLR(%)=100×(FW–FWt)/FW.

### Measurement of stomatal aperture

Stomatal aperture was determined by making epidermal impressions of the adaxial leaf surface. Leaf impressions were prepared with polyvinylsiloxane-based high precision President Light Body impression material (Coltène/Whaledent AG, Altstätten, Switzerland) modified from Geisler *et al.* (2000). Leaves were excised from plants and immediately placed onto the impression material. After solidification of the impression material, leaves were gently removed, leaving behind epidermal imprints. Transparent and colourless nail polish was then applied to the imprints and allowed to dry. The dried nail polish peels were carefully stripped and placed on a microscope slide. Impressions of leaf stomata were examined under an Axiophot light microscope (Zeiss, Oberkochen, Germany) and images were captured with an AxioCam ERc5S digital camera (Zeiss). The stomatal aperture was measured using AxioVision software release 4.8.2 (Zeiss).

### Quantitative real-time PCR

Per treatment, nine randomly selected *Arabidopsis* seedlings were harvested, pooled, and ground to a fine powder in liquid nitrogen. Total RNA was extracted using an RNeasy Plant Mini Kit (Qiagen, Valencia, CA, USA) and subjected to a treatment with RNase-free DNase I (Qiagen) following the manufacturer’s instructions. The extracted RNA served as template for the synthesis of single-stranded cDNA templates with the QuantiTect Reverse Transcription Kit (Qiagen) according to the manufacturer’s instructions. Quantitative real-time PCR (qRT-PCR) was performed using the SYBR Green Supermix with a CFX96 Real-Time PCR Detection System (Bio-Rad Laboratories, Hercules, CA, USA). All qRT-PCR assays were performed as follows: 95 °C for 3min, 40 cycles of 95 °C for 10 s, 55 °C for 30 s. At the end of the PCR, the temperature was increased from 55 °C to 95 °C to generate the melting curve. The expression of the following genes was measured: *1-aminocyclopropane-1-carboxylate oxidase* (*ACO*) (*At2g19590*), *alcohol dehydrogenase* (*ADH*) (*At1g77120*), *ethylene receptor* (*ETR2*) (*At3g23150*), *major intrinsic family protein* (*NIP2;1*) (*At2g34390*), *phosphofructokinase-6* (*PFK6*) (*At4g32840*), *pyruvate decarboxylase-1* (*PDC1*) (*At4g33070*), *pyruvate decarboxylase-2* (*PDC2*) (*At5g54960*), *sucrose synthase-1* (*SUS1*) (*At5g20830*). and *sucrose synthase-4* (*SUS4*) (*At3g43190*), respectively. The primer pairs used for qRT-PCR are shown in Supplementary Table S1 available at *JXB* online. The relative changes in gene expression were calculated by the 2^–ΔΔCt^ method (Livak and Schmittgen, 2001). The expression levels of genes of interest were normalized to the expression level of the gene *actin-2* (*ACT2*) (*At3g18780*). As controls, the relative expression levels of the reference genes *polyubiquitin-10 (UBQ10*) (*At4g05320*) and *β-6-tubulin* (*TUB6*) (*At5g12250*) were evaluated.

### Visualization of superoxide radicals

Superoxide radicals were detected by staining seedlings with nitroblue tetrazolium (NBT) solution according to Ramel *et al.* (2009) with minor modifications. Seedlings, including roots, were taken from *in vitro* culture dishes and inserted into a 0.1% (w/v) NBT staining solution containing 50mM phosphate buffer (pH 7.8) and 10mM NaN_3_. Seedlings were vacuum-infiltrated for 2min and kept for 30min in the dark at room temperature. Stained seedlings were then bleached in 70% (v/v) ethanol at 80 °C and photographs were taken.

## Results

### Culturing on a low concentration of Gelrite induced HH in *Arabidopsis thaliana* seedlings

**Fig. 1.**
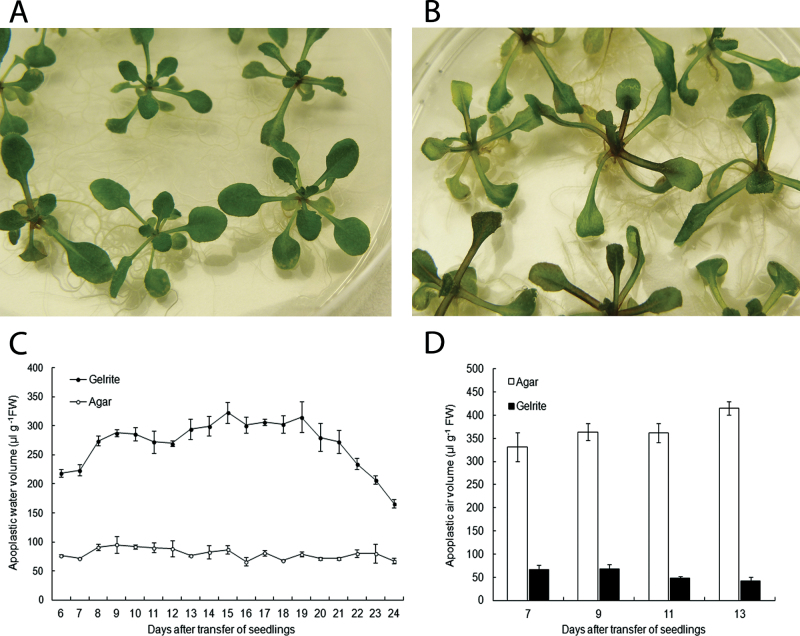
Development of HH in Arabidopsis seedlings. Seedlings were cultured for 7 d on 0.7% agar, and then transferred to fresh medium with 0.7% agar or to 0.2% Gelrite. Photographs were taken 10 d after transfer of the seedlings. (A) Seedlings cultured on 0.7% agar exhibited no symptoms of HH. (B) Seedlings cultured on 0.2% Gelrite developed HH. (C) Water accumulation in leaf apoplast of seedlings grown on 0.7% agar or 0.2% Gelrite. Each value is the mean of three samples of nine seedlings ±SE. (D) Apoplastic air volume (μl g^−1^ FW) in leaves of hyperhydric seedlings from Gelrite and non-hyperhydric seedlings from agar. Each value is the mean of three samples of three randomly selected seedlings ±SE.

*Arabidopsis thaliana* seedlings were stratified for 3 d at 4 °C, cultured for 7 d at 21 °C on 0.7% agar, and then transferred to 0.2% Gelrite to induce HH. Control seedlings were transferred to fresh 0.7% agar. It was noticed that during the first week after transfer, seedlings on Gelrite grew slightly faster than seedlings on agar. After 7 d of culture on media with Gelrite, seedlings showed a hyperhydric phenotype with thick, translucent leaves and elongated petioles (Fig. 1B). The control seedlings that were kept on agar media developed no symptoms of HH (Fig. 1A). Fifteen days after transfer, hyperhydric seedlings exhibited signs of chronic stress, such as anthocyanin production, chlorosis, and leaf necrosis. This indicates that stress levels increased during the later stages of HH. After 25 d, all seedlings grown on media with Gelrite had died, whereas the control seedlings cultured on agar were still alive and apparently healthy.

### Apoplastic water and air volumes in hyperhydric seedlings

Changes in the apoplastic water volume during the development of HH were followed during a 20 d period, starting 6 d after transfer of seedlings to Gelrite. Water located in the leaf apoplast was extracted by centrifugation at low speed. To confirm that the extracted water was primarily originating from the apoplast, the cytoplasmic contamination in the collected fluid was assessed by measuring MDH activity (Supplementary Fig. S1 at *JXB* online). At the centrifugation speed used for water collection (3000 *g*), the cytoplasmic contamination was low, so most of the water originated from the apoplast. Figure 1C shows that hyperhydric seedlings cultured on Gelrite accumulated substantially more water in the apoplastic space than agar-grown seedlings. The volume of apoplastic water in seedlings from Gelrite increased along with the development of HH symptoms. From 8 d after the transfer to Gelrite onwards, the water volume in the apoplast of hyperhydric seedlings remained more or less constant, indicating that the apoplast was saturated. The volume of water in the leaf apoplast of hyperhydric seedlings was about three times higher than that of non-hyperhydric seedlings. After 20 d on Gelrite, a sharp decline in the apoplastic water volume in hyperhydric seedlings was observed, caused by necrosis of leaf tissues.

Experiments were carried out to determine whether the volume of apoplastic water in hyperhydric leaves increased at the expense of the air volume in the apoplast. The apoplastic air volume was estimated by a vacuum infiltration method using a pycnometer and was measured in seedlings on days 7, 9, 11, and 13 after transfer. Compared with non-hyperhydric seedlings, the volume of apoplastic air was markedly smaller in hyperhydric leaves at all four time points (Fig. 1D). In control and hyperhydric seedlings, air occupied 85% and 15% of the total apoplastic volume, respectively. The total volume of the apoplast was calculated by adding the apoplastic water and air volumes. These data reveal that the increase of water in the apoplast was accompanied by a very strong reduction in apoplastic air volume.

### Apoplastic water and air volumes in hyperhydric statice plantlets

**Fig. 2.**
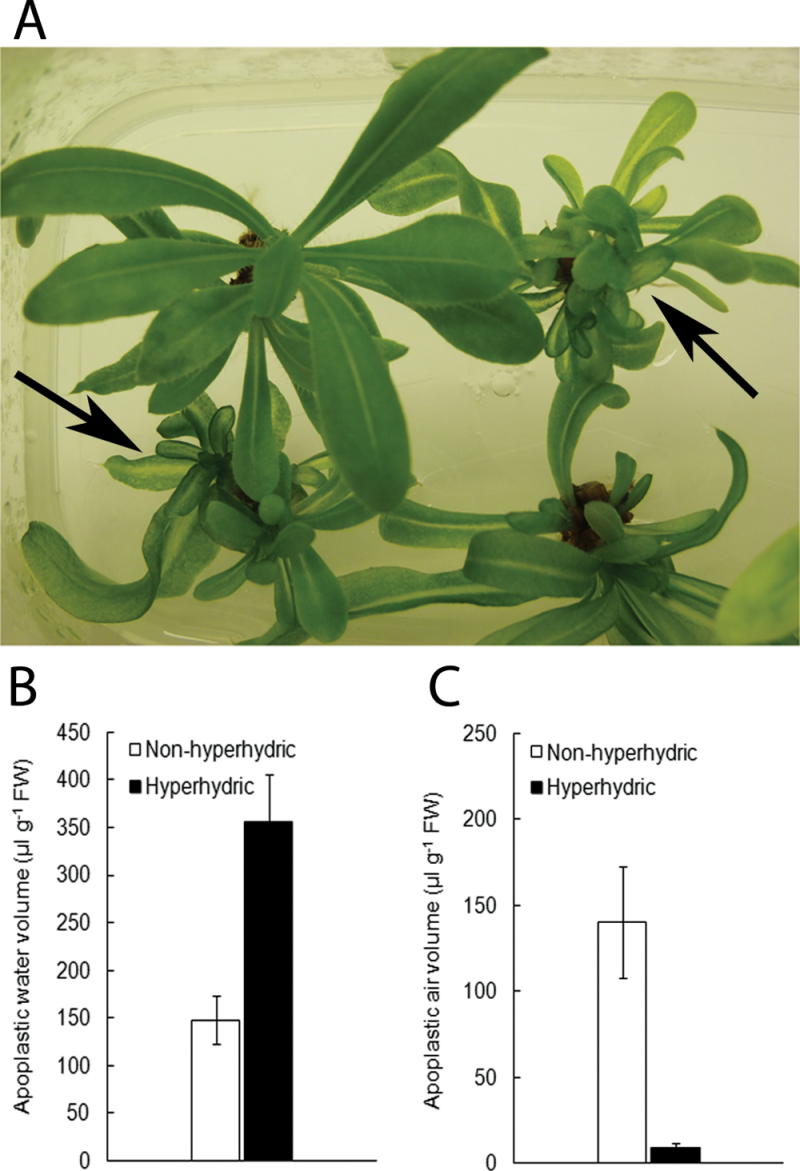
Development of HH in statice. (A) Statice plantlets cultured on 0.2% Gelrite developed HH, but often only several leaves were affected. Hyperhydric shoots are indicated by arrows. (B and C) Comparison of apoplastic water and apoplastic air volumes (μl g^–1^ FW) in leaves of hyperhydric and non-hyperhydric statice plantlets. Each value is the mean of three samples of three randomly selected plantlets± SE.

Experiments were also carried out to examine whether flooding of the apoplastic space during HH occurs in other species as well. In statice (*L. sinuatum*), HH was also induced by growing shoots on 0.2% Gelrite. HH developed, but less abundantly than in *Arabidopsis* (Fig. 2A). Nonetheless, consistent with *Arabidopsis*, the same relationship between apoplastic water and air volumes was found in hyperhydric statice shoots. The volume of water in the leaf apoplast of hyperhydric shoots had increased >2-fold compared with non-hyperhydric shoots (Fig. 2B). Apoplastic air volume was reduced from ~50% of the apoplastic volume in non-hyperhydric shoots to <5% in hyperhydric shoots (Fig. 2C). Similar results were obtained for hyperhydric shoots of apple and crambe (data not shown).

### Hyperhydric seedlings suffer from hypoxia

**Fig. 3.**
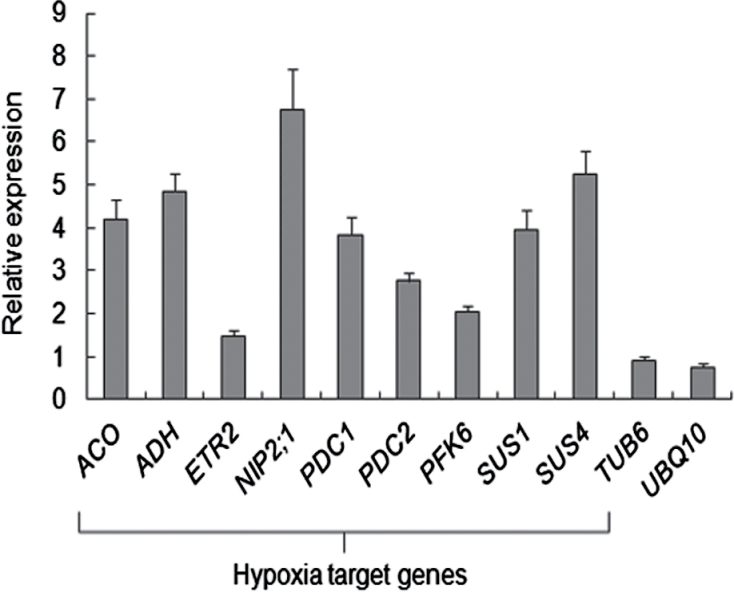
Expression of hypoxic-responsive genes in hyperhydric *Arabidopsis* seedlings. After 7 d growth on 0.7% agar, seedlings were transferred to fresh 0.7% agar or to 0.2% Gelrite. Total RNA was extracted from complete seedlings 10 d after transfer. Relative expression levels of the indicated hypoxia-target genes were quantified by qRT-PCR and normalized to *Act2* levels. As controls, expression levels of the housekeeping genes *UBQ10* and *TUB6* were assessed. Each value is the mean ±SE of three biological and six technical replicates, and values are presented as expression fold change (Gelrite versus agar).

It was assumed that the lack of air in the apoplast of hyperhydric plants hampers the exchange of gases between symplast and its environment and, as a consequence, also the supply of oxygen to the symplast. Therefore, the expression of a set of hypoxia-responsive genes was analysed by qRT-PCR: *ACO*, *ADH*, *ETR2*, *NIP2;1*, *PDC1*, *PDC2*, *PFK6*, *SUS1*, and *SUS4*. It has been reported that the expression of these genes is induced in *Arabidopsis* subjected to hypoxic conditions (Liu *et al.*, 2005). Expression levels in seedlings were assessed 10 d after transfer to media containing either agar or Gelrite. All genes, except *ETR2*, showed at least 2-fold up-regulation in Gelrite-grown seedlings (Fig. 3). This strongly indicates that hyperhydric seedlings were under hypoxia. Hypoxic conditions can be responsible for the generation of reactive oxygen species (ROS). NBT staining for the presence of the superoxide radical showed that indeed hyperhydric seedlings produced higher levels of ROS than non-hyperhydric seedlings (Supplementary Fig. S2 at *JXB* online).

### Stomatal closure in hyperhydric plants

**Fig. 4.**
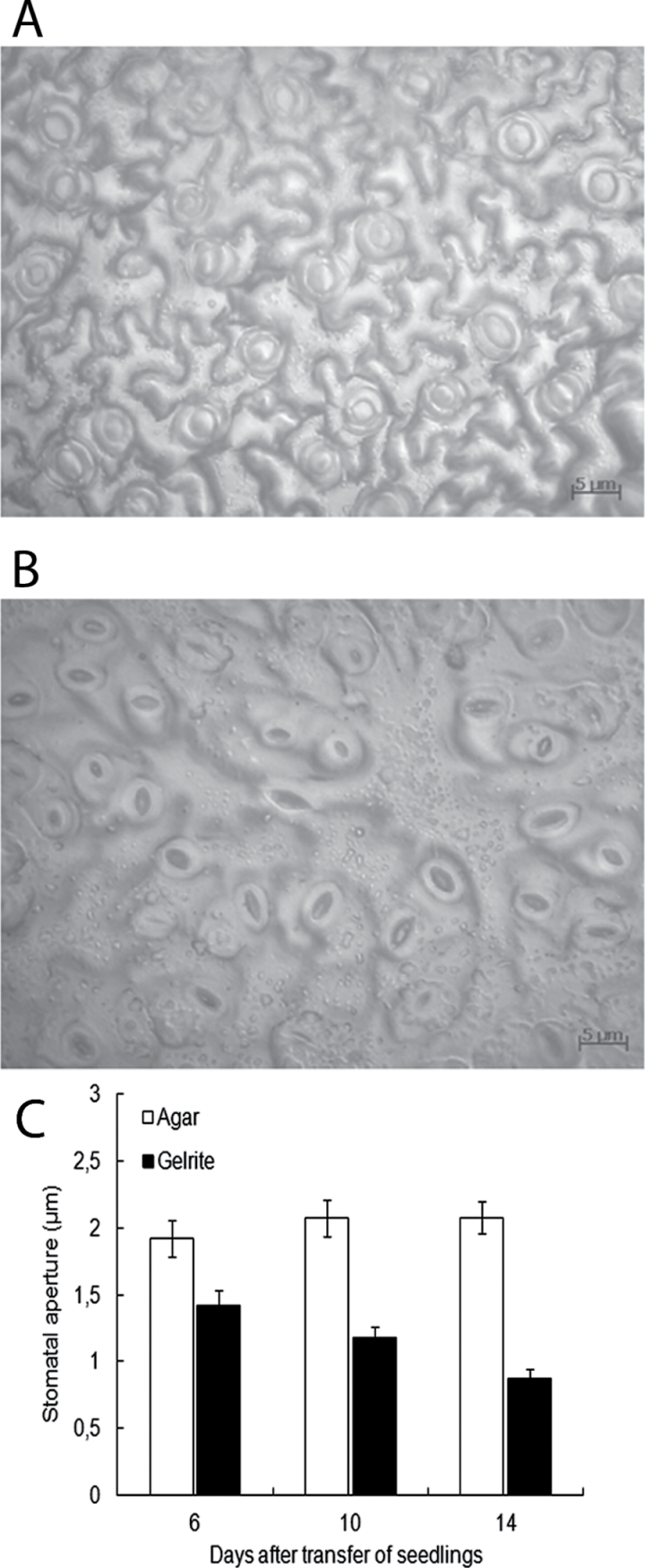
Influence of HH on stomatal aperture in *Arabidopsis* seedlings. Seedlings were cultured for 7 d on 0.7% agar, and then transferred to fresh 0.7% agar or 0.2% Gelrite. Microscopic photographs of adaxial leaf impressions of (A) hyperhydric and (B) non-hyperhydric seedlings were taken 10 d after transfer of seedlings. (C) Stomatal apertures in leaves of hyperhydric and non-hyperhydric seedlings measured 6, 10, and 14 d after transfer, respectively. Each value is the mean of determinations of >90 stomata on leaves of nine randomly selected seedlings ±SE. (This figure is available in colour at *JXB* online.)

HH has been shown to lead to abnormal and malfunctioning stomata (Apostolo and Llorente, 2000). Stomatal characteristics were explored in hyperhydric tissues of both *Arabidopsis* and statice. Stomatal aperture was measured in *Arabidopsis* seedlings grown for 6, 10, and 14 d on Gelrite media. For comparison, stomatal aperture was also determined in agar-grown seedlings (Fig. 4A). It was observed that stomata in the leaves of hyperhydric seedlings were partially or fully closed (Fig. 4B). Figure 4C shows that the opening of stomata declined with the development of HH symptoms. After 14 d on Gelrite, stomatal aperture in seedlings was ~58% smaller to that of agar-grown seedlings. No difference in stomatal density between hyperhydric and non-hyperhydric seedlings was detected. A similar stomatal response was observed in hyperhydric leaves of statice (Supplementary Fig. S3 at *JXB* online). The stomatal aperture in hyperhydric statice shoots decreased to ~45% of that found in non-hyperhydric plantlets. Taken together, these results implicate a negative relationship between stomatal aperture and HH.

### Effect of gelling agent on *ex vitro* acclimatization of seedlings

**Fig. 5.**
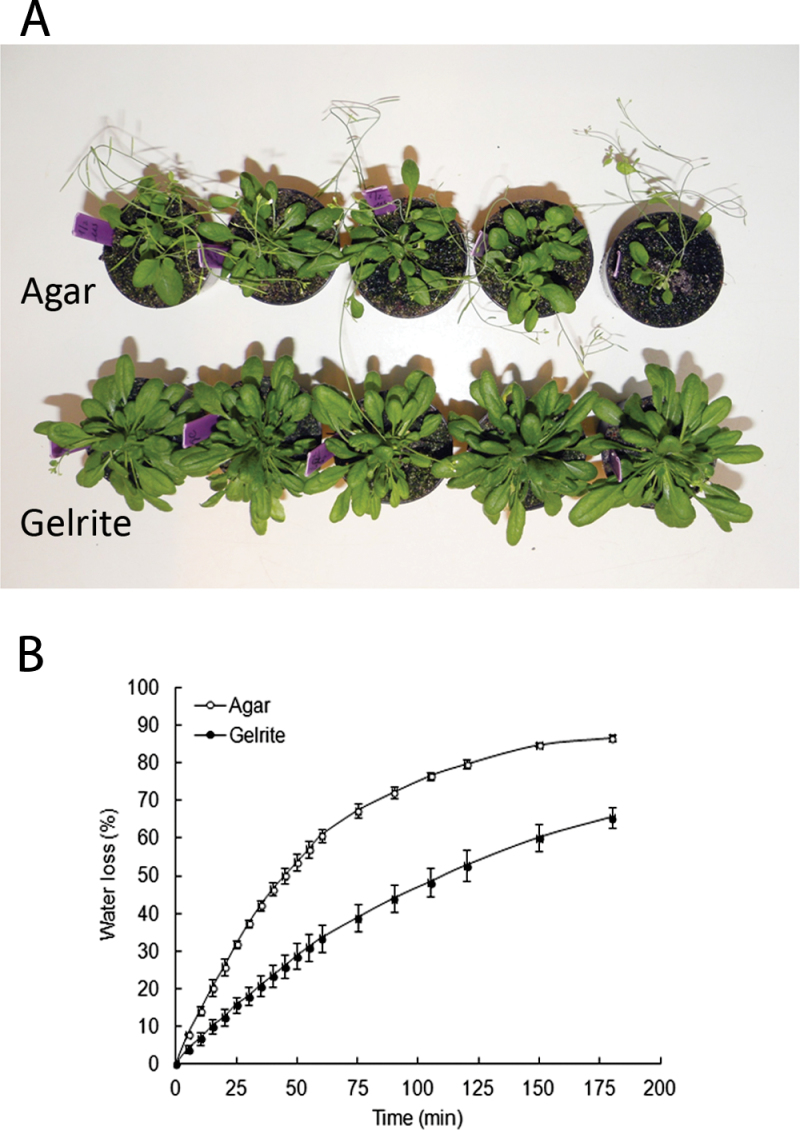
Effect of gelling agent on the growth of Arabidopsis seedlings after transfer to soil. (A) After 7 d of growth on 0.7% agar, seedlings were transferred to 0.7% agar or 0.2% Gelrite. Ten days after transfer, seedlings were transplanted to soil, grown for 30 d, and photographed. Note that the Gelrite-grown seedlings are much larger. (B) Water loss rates of seedlings cultured for 10 d after transfer on 0.7% agar or 0.2% Gelrite. Each value is the mean of determinations on three samples of leaves taken from nine randomly selected seedlings ±SE.

To examine whether hyperhydric and non-hyperhydric seedlings vary in their ability to acclimatize to *ex vitro* conditions, 1-week-old seedlings were cultured for 10 d on either 0.7% agar or 0.2% Gelrite before being planted into soil. The seedlings grown on media with Gelrite for 10 d showed moderate symptoms of HH, but had not yet developed symptoms of chronic stress, such as chlorosis. After transfer to soil, Gelrite-grown seedlings fully recovered from HH and showed a better performance than seedlings from agar (Fig. 5A). The seedlings from Gelrite developed larger rosettes with more and longer leaves than seedlings from agar. The water retention capacity of seedlings may affect the ability to adapt to the *ex vitro* conditions. Seedlings grown on Gelrite media had a lower water loss rate under *ex vitro* conditions and maintained more water than seedlings from agar (Fig. 5B). Extending the culture period of seedlings on Gelrite media before transplantation had a negative effect on *ex vitro* survival of seedlings. Thus, the improvement of *ex vitro* acclimatization depended on a low degree of HH.

## Discussion

Most studies on HH dealt with prevention and with symptoms at anatomical, biochemical, and occasionally molecular levels, but only few with the underlying mechanisms. The subcellular localization of the extra water in hyperhydric plants has never been critically examined, with the exception of research in *Gypsophila*. In this species, it was found that in hyperhydric plants more water was located in the intercellular spaces (Gribble, *et al.*, 1996, 1998). The authors supposed that the volume of apoplastic water increased at the expense of the apoplastic air (Gribble *et al.* 1998, 2003). However, the volumes of apoplastic water and air were not quantified. In the present study, the volumes of water and air in the apoplast of hyperhydric and non-hyperhydric tissues have been adequately measured for the first time. In hyperhydric plants, the volume of apoplastic water increased dramatically. The excessive accumulation of water in the apoplast caused a drastic reduction in apoplastic air in *Arabidopsis* and in the other species that were examined (statice Fig. 2; apple and crambe, data not shown). It was calculated that the volume of air in *Arabidopsis* leaves (determined as a percentage of the volume of the apoplast) decreased from 85% in non-hyperhydric plants to 15% in hyperhydric ones.

Water in the apoplast was extracted by mild centrifugation, and the apoplastic air space was estimated using a vacuum infiltration technique with a pycnometer. Possible constraints of these procedures are that water and/or air remained trapped within the apoplast, resulting in underestimation of the total apoplastic volume, and that due to damage of cells their content was also extracted by centrifugation, leading to overestimation. However, both methods provided realistic estimates in the non-hyperhydric leaves, which were within the range of volumes observed in other plant species (Lohaus *et al.*, 2001). In most previous studies on HH, the severity of the disorder was assessed by visual evaluation of morphological abnormalities, a method that is inherently subjective. The volumes of water and air in the apoplast may be more appropriate parameters to monitor the extent of HH.

### Causes of the flooding of the apoplast

Evidently, the apoplast becomes flooded when there is too much entry and/or too little removal of water. With respect to entry, the occurrence of HH increases when the availability of water increases. The type and concentration of the gelling agent used in the nutrient medium have an effect on the water availability. Gelrite was used here at an intermediate concentration (0.2%) to induce HH in both *Arabidopsis* and statice. Gelrite has been shown to generate HH in various plant species (Turner and Singha, 1990; Franck *et al.*, 2004; Ivanova and Van Staden, 2011). Gelrite may increase the availability of water by allowing a higher uptake of water. Chelators excreted by plants may dissolve the Gelrite gel locally so that it resembles culture on liquid medium. In this respect, it should be noted that a Gelrite gel liquefies instantaneously when drops of a solution of the chelating agent EDTA are added (G. de Klerk, unpublished observation). The reason for this is that Gelrite requires bivalent cations for solidification and EDTA captures these bivalent cations. After some days of culture with *Arabidopsis* seedlings, a gel solidified with Gelrite releases much more water than a gel solidified with agar (G. de Klerk and J. Gao, unpublished results). It has been suggested that the physical structure of Gelrite brings about an increase in absorption by shoots of certain compounds, such as cytokinins, which in turn cause HH (Franck *et al.*, 2004; Ivanova and Van Staden, 2011). This is, however, doubtful considering the finding that the uptake rate of radiolabelled benzyladenine from agar and Gelrite media is the same (Bornman and Vogelmann, 1984). The removal of water from the apoplast by transpiration is limited due to the high relative humidity in the growth container (~99.5%, Chen, 1994). When the humidity is reduced by ventilation or by bottom cooling, HH considerably decreases (Park *et al.*, 2004; Saher *et al.*, 2005; Ivanova and Van Staden, 2010). Finally, it should be noted that it is not only the entry and removal of water that determine flooding of the intercellular spaces, but that the characteristics of the cell walls bordering the intercellular spaces are also crucial. The most likely mechanism to keep the spaces from being flooded is that plants maintain a hydrophobic monolayer on the surfaces of adjacent intercellular spaces, but this is still debated (Woolley, 1983; Raven, 1996).

Ethylene accumulation in the headspace of growth containers has been frequently associated with HH (Matsurbara *et al.*, 1991; Franck et al, 2004; Lai *et al.*, 2005). It was found here that the expression of *ACO*, encoding an enzyme involved in ethylene biosynthesis, was elevated in hyperhydric seedlings. It has been shown that silver nitrate, a compound that inhibits ethylene action, reduces HH in sunflower (Mayor *et al.*, 2003). If ethylene induces HH, ethylene-insensitive *Arabidopsis* mutants should not develop HH. However, on Gelrite media, an ethylene-insensitive mutant (*etr1-1*) developed HH similar to the wild type (N. van den Dries, unpublished observations). Consequently, there seems to be no role for ethylene in the development of HH in *Arabidopsis* seedlings cultured on Gelrite. The nitrogen source in the culture medium also plays a role in HH. Ivanova and Van Staden (2009) showed that HH was reduced in *Aloe polyphylla* by decreasing the ratio of ammonium to nitrate ions in the medium. Various other medium components increase the incidence of HH, such as a high sucrose concentration (Zimmerman and Cobb, 1989). Possibly medium components influence HH by changing the characteristics of the cell wall adjacent to the intercellular spaces, making it less hydrophobic, as discussed at the end of the previous paragraph.

### Consequences of flooding of the apoplast

Flooding of the apoplast is most probably the physiological basis of hyperhydric abnormalities, because it hampers gas exchange between the symplast and the surrounding atmosphere. The reduced gas exchange in hyperhydric tissues was evidenced by the occurrence of hypoxic stress. Given that hypoxia elicits oxidative stress (Vergara *et al.*, 2012), many deleterious effects in hyperhydric tissues are probably related to oxidative damage. Oxidative stress in hyperhydric plants has been reported for various species (Olmos *et al.*, 1997; Saher *et al.*, 2004; Chakrabarty *et al.*, 2005). To reduce the damaging effects of oxidative stress, the activity of antioxidant enzymes is often enhanced in hyperhydric plants (Dewir *et al.*, 2006; Balen *et al.*, 2009). The reduced gas exchange during HH may also result in the accumulation of gaseous compounds inside the cells, such as ethylene, methyl jasmonate, and methyl salicylate. As a matter of fact, hyperhydric plants experience similar stress to flooded plants, but in hyperhydric plants the flooding occurs within the tissues. Accumulation of ethylene has also been observed in flooded plants (Voesenek *et al.*, 1993). In addition, hyperhydric seedlings displayed elongated petioles, and it has been shown that ethylene can trigger petiole elongation in *Arabidopsis* (Millenaar *et al.*, 2005).

Various studies have reported malformed and non-functional stomata in hyperhydric leaves (Werker and Leshem, 1986; Apostolo and Llorente, 2000; Picoli *et al.*, 2001). In the present study a reduction in stomatal aperture was observed in hyperhydric leaves of both *Arabidopsis* and statice. Stomatal closure during HH has also been observed in shoots of carnation (Ziv and Ariel, 1994). There are several possible causes of this stomatal closure. It may be caused by enhanced stress levels in HH plants, for example by waterlogging of roots, which induces stomatal closure (Atkinson *et al.*, 2008; Else *et al.*, 2009; Rodriguez-Gamir *et al.*, 2011). It was noticed that stomatal closure in hyperhydric seedlings occurred simultaneously with the increase of water in the apoplast. In agreement with this, Sibbernsen and Mott (2010) demonstrated that flooding of the leaf apoplast with water by microinjection or by vacuum infiltration resulted in rapid stomatal closure in different plant species. The closure of the stomata evidently reduces transpiration and thereby contributes to the flooding of the apoplast, but it remains to be determined whether this is a crucial factor in the development of HH. Mesophyll cells in the apoplastic space are capable of perceiving signals from bacteria to trigger stomatal closure (Melotto *et al.*, 2008; Zeng *et al.*, 2010). Although HH plants are grown in a sterile environment, a similar signalling pathway leading to stomatal closure might be activated by flooding of the apoplast. There is a great deal of evidence supporting the existence of cross-talk between abiotic and biotic stress responses (Torres and Dangl, 2005; Fujita *et al.*, 2006).

### Planting out hyperhydric seedlings

Growing *Arabidopsis* seedlings for a short period (10 d) on media solidified with 0.2% Gelrite increased the performance of the seedlings after planting in soil. Seedlings transplanted to *ex vitro* conditions are exposed to drought stress due to the dramatic drop in humidity. Possibly the large water volume in the apoplast of Gelritre-grown seedlings acts as an additional water reserve during *ex vitro* acclimatization. The reduced stomatal aperture in Gelrite-grown seedlings may also provide protection against dehydration. Delarue *et al.* (1997) observed that, during dehydration treatments, the wilting process was delayed in the hyperhydric *Arabidopsis* mutant (*cri1*). It was found that for successful acclimatization, it was critical that seedlings were transferred to soil before tissues were irreversibly damaged by HH-related stress.

### Conclusions

**Fig. 6.**
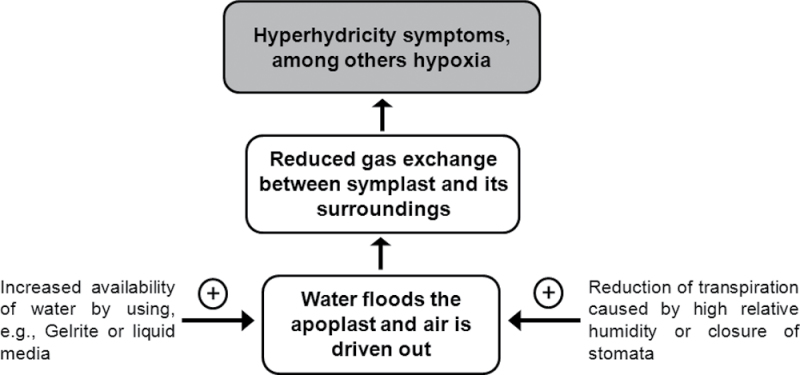
Schematic diagram showing the crucial role of water in the apoplast in the development of HH.

In the present work, the volumes of apoplastic water and air in hyperhydric and non-hyperhydric plants were quantified for the first time. It was demonstrated that the excess water accumulated in the apoplast of hyperhydric plants and caused a dramatic reduction in apoplastic air volume. Additionally, it was evidenced that flooding of the apoplastic space impaired gas exchange by the symplast as shown by the occurrence of hypoxia. The imbalance between water and air in the apoplastic space is a critical factor in HH (Fig. 6). The high availability of water in the nutrient medium, especially when Gelrite or liquid media are used, and the high relative humidity in the growth container, which reduces transpiration, are the main causes of HH. It remains to be clarified how cytokinins and the ammonium/nitrate ratio influence HH. A molecular study on HH could provide more insight into this disorder and may lead to new treatments to eliminate it. The present study showed that the development of HH can be closely monitored in *Arabidopsis* by measuring volumes of water and air in the apoplast, thereby providing an excellent model species to study the molecular basis of HH.

## Supplementary data

Supplementary data are available at *JXB* online.

Table S1. Primer sequences used for quantitative real-time PCR analysis.

Figure S1. Effect of centrifugation force on cytoplasmic contamination in apoplastic water.

Figure S2. Visualization of superoxide radicals by nitroblue tetrazolium staining.

Figure S3. Influence of HH on stomatal aperture in statice plantlets.

Supplementary Data
