# Corrigendum

**DOI:** 10.1093/jxb/eru497

**Published:** 2015-01-17

**Flooding of the apoplast is a key factor in the development of hyperhydricity**

**Niels van den Dries, Sergio Giannì, Anna Czerednik, Frans A. Krens and Geert-Jan M. de Klerk**

*Journal of Experimental Botany* 2013, 64, 5221–5230. doi:10.1093/jxb/ert315

In the above article, the formulas for estimating the volumes of water and air in the apoplast were presented incorrectly. The corrected formulas are given below. The data in this article were not affected, because correct formulas were used in all calculations. The authors would like to apologize for possible inconveniences these errors may have caused.

The apoplastic water volume (V_water_) in µl g^-1^ FW was calculated using the corrected formula:

$${\text{V}}_{\text{water}}=\;\left[\left(\text{FW}–{\text{W}}_{\text{ac}}\right)/\rho {\text{H}}_{2}\text{O}\right]/\text{FW }$$

Where FW = fresh weight of leaves, W_ac_ = weight of leaves after centrifugation and ϱH_2_O = water density (we took the water density equal to 1g ml^-1^).

The apoplastic air volume (V_air_) in µl g^−1^ FW was calculated using the corrected formula:

$${\text{V}}_{\text{air}}=\left[\left({\text{W}}_{\text{av}}–{\text{W}}_{\text{bv}}\right)/\varrho {\text{H}}_{2}\text{O}\right]/\text{FW }$$

Where: W_av_ = weight of pycnometer together with leaves and distilled water after vacuum infiltration, W_bv_ = weight of pycnometer together with leaves and distilled water before vacuum infiltration, FW = fresh weight of leaves and ϱH_2_O = water density (we took the water density equal to 1g/ml).

