# Supplementary material for: Flooding of the apoplast is a key factor in the development of hyperhydricity
Source: J Exp Bot. 2013 Oct 11;64(16):5221–30. doi: 10.1093/jxb/ert315 (PMC3830496; doi:10.1093/jxb/ert315)
Supplement: Supplementary Data [file supp_64_16_5221__index.html]

Flooding of the apoplast is a key factor in the development of hyperhydricity — Flooding of the apoplast is a key factor in the development of hyperhydricity — Supplementary Data 

# Flooding of the apoplast is a key factor in the development of hyperhydricity

## Supplementary Data

Data files

**Files in this Data Supplement:**

- Supplementary Data - Supplementary Data
