# Supplementary material for: Flooding of the apoplast is a key factor in the development of hyperhydricity
Source: J Exp Bot. 2013 Oct 11;64(16):5221–30. doi: 10.1093/jxb/ert315 (PMC3830496; doi:10.1093/jxb/ert315)
Supplement: Supplementary Data [file supp_ert315_jexbot104810_file001.pdf]

Title: Flooding of the apoplast is the key factor in the development of hyperhydricity.

Authors: Niels van den Dries, Sergio Giannì, Anna Czerednik, Frans A. Krens and Geert-Jan M. de Klerk

Supplementary Data

Table S1

Gene ID	AGI ID	Forward primer (5'→3')	Reverse primer (5'→3')
ACO	*At2g19590*	CTCAGCAAGACGATGGATGA	TTGGACCAGAAAAGGCATTC
ACT2	*At3g18780*	GGTAACATTGTGCTCAGTGGTGG	AACGACCTTAATCTTCATGCTGC
ADH	*At1g77120*	GAATCGCTGGTGCTTCTAGG	CTCAGCGATCACCTGTTGAA
ETR2	*At3g23150*	TTGTGCTACTGCGATTACGC	CAACTTCACGACCAAGCTCA
NIP2;1	*At2g34390*	TTACTGTCTCGGCCACCTCT	CCAAGGTTGATCCGATGACT
PDC1	*At4g33070*	GGTGGTCCTAAGTTGCGTGT	CTGCTCCCCAATAAGTTCCA
PDC2	*At5g54960*	CTAATAGTCGTCCCCCAAATCC	GAACGGTGAAGACACTACCAAA
PFK6	*At4g32840*	CGATCTCCCCACTTATCCAA	GGCCTGCACGTCTAAAATGT
SUS1	*At5g20830*	CGCCGTTACCTTGAAATGTT	CTTCAAACACCGGAACCACT
SUS4	*At3g43190*	ACAAACTCAACGGGCAATTC	AAAGAGCAGGCTGCACAAAT
TUB6	*At5g12250*	ACCACTCCTAGCTTTGGTGATCTG	AGGTTCACTGCGAGCTTCCTCA
UBQ10	*At4g05320*	CACACTCCACTTGGTCTTGCGT	TGGTCTTTCCGGTGAGAGTCTTCA

Supplementary Figure S1

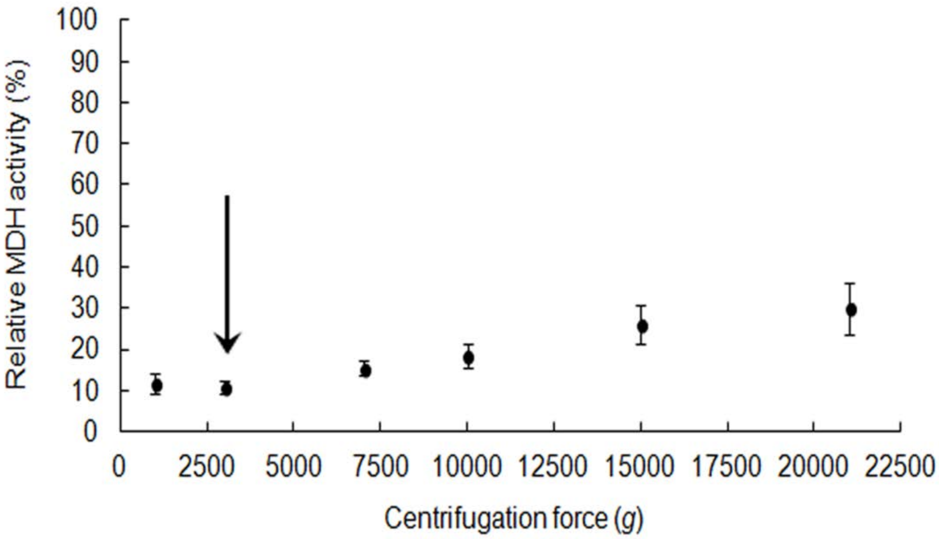

Supplementary Fig. S1

Effect of centrifugation force on cytoplasmic contamination in extracted apoplastic water. Contamination of apoplastic water with cytoplasmic components was assessed by measuring the activity of malate dehydrogenase (MDH) at increasing centrifugation forces. The MDH activity in the extracted water was expressed as the percentage of total MDH activity. The standard centrifugation force applied for extracting apoplastic water from leaves was 3000*g* and is indicated by an arrow. Data points represent means ± SE from three independent measurements.

Supplementary Figure S2

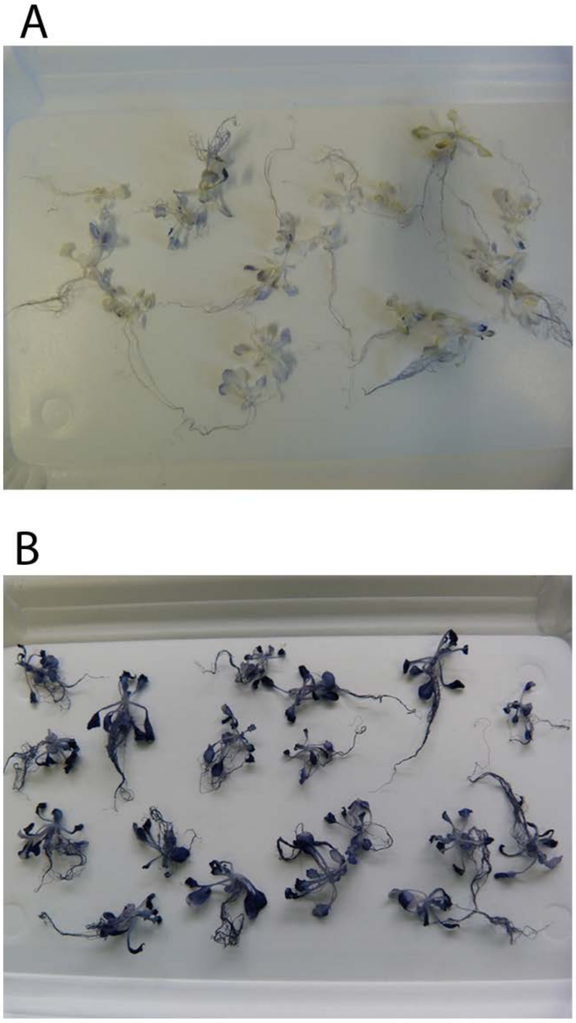

Supplementary Fig. S2

Visualization of superoxide radicals by nitroblue tetrazolium (NBT) staining. Representative photographs showing the presence of superoxide radicals in (A) non-hyperhydric and (B) hyperhydric Arabidopsis seedlings. The presence of superoxide radicals is visualized by a blue precipitate.

Supplementary Figure S3

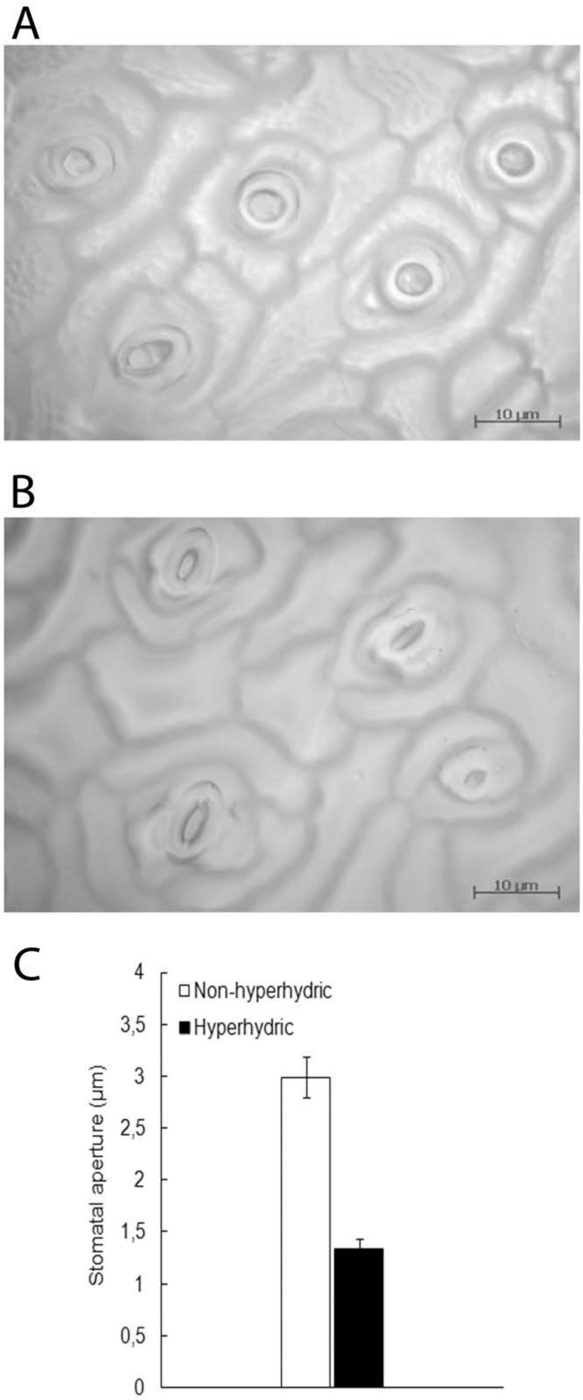

Supplementary Fig. S3

Influence of HH on stomatal aperture in statice plantlets. Microscopic photographs of adaxial leaf impressions of (A) hyperhydric and (B) non-hyperhydric statice plantlets. (C) Stomatal apertures in leaves from hyperhydric and non-hyperhydric statice plantlets. Each value is the mean of determinations of more than 90 stomata on leaves of three randomly selected plantlets ± SE.
